# Conflict Dynamics in Scale-Free Networks with Degree Correlations and Hierarchical Structure

**DOI:** 10.3390/e24111571

**Published:** 2022-10-31

**Authors:** Eduardo Jacobo-Villegas, Bibiana Obregón-Quintana, Lev Guzmán-Vargas, Larry S. Liebovitch

**Affiliations:** 1 Facultad de Ciencias, Universidad Nacional Autonoma de Mexico, Ciudad de Mexico 04510, Mexico; 2 Unidad Interdisciplinaria en Ingenieria y Tecnologias Avanzadas, Instituto Politecnico Nacional, Av. IPN No. 2580, L. Ticomán, Ciudad de Mexico 07340, Mexico; 3 Department of Physics, Queens College, City University of New York, New York, NY 11367, USA

**Keywords:** dynamic of conflicts, scale-free networks, hierarchical networks, cooperation

## Abstract

We present a study of the dynamic interactions between actors located on complex networks with scale-free and hierarchical scale-free topologies with assortative mixing, that is, correlations between the degree distributions of the actors. The actor’s state evolves according to a model that considers its previous state, the inertia to change, and the influence of its neighborhood. We show that the time evolution of the system depends on the percentage of cooperative or competitive interactions. For scale-free networks, we find that the dispersion between actors is higher when all interactions are either cooperative or competitive, while a balanced presence of interactions leads to a lower separation. Moreover, positive assortative mixing leads to greater divergence between the states, while negative assortative mixing reduces this dispersion. We also find that hierarchical scale-free networks have both similarities and differences when compared with scale-free networks. Hierarchical scale-free networks, like scale-free networks, show the least divergence for an equal mix of cooperative and competitive interactions between actors. On the other hand, hierarchical scale-free networks, unlike scale-free networks, show much greater divergence when dominated by cooperative rather than competitive actors, and while the formation of a rich club (adding links between hubs) with cooperative interactions leads to greater divergence, the divergence is much less when they are fully competitive. Our findings highlight the importance of the topology where the interaction dynamics take place, and the fact that a balanced presence of cooperators and competitors makes the system more cohesive, compared to the case where one strategy dominates.

## 1. Introduction

In recent years, the study of the dynamics among actors, which may be people, species, neuronal regions, etc., located on complex networks has allowed for a quantitative description of various systems in different areas of science, such as ecology [1], biochemistry [2], neuroscience [3], epidemiology [4], and the social sciences [5,6]. The mathematical formalism of such descriptions has not only made quantitative descriptions to verify previous empirical observations but has sometimes provided novel predictions [7,8,9,10]. How the values of the nodes change in time, and the dynamics of these networks, depends on how the nodes are connected to each other, that is, the topology of the network [9,11,12,13]. Studies of network dynamics first concentrated on the degree distribution and the number of connections of each node [14,15]. In order to capture the network properties observed in real biological and social networks [1,2,3,4,5], a more realistic approach was needed because nodes are not randomly connected to other nodes. The topology of these networks is more structured with the existence of “hubs” or “clusters” that have many more than an average number of connections to other nodes [16,17].

The detailed structure of these hubs or clusters plays an important role in dynamics [9,11,12,13]. In scale-free topologies, the topology of the degree distribution at one scale in the network is self-similar to the degree distribution at other scales, for example, when the degree distribution is a power law. Network models with these distributions have proved valuable in understanding the dynamical processes in digital communications [18], artificial intelligence [19,20], optimization [21], transportation [22], neurology [23], epidemiology [24], wireless sensor networks [25], biology [26,27], and the evolution of cooperative behaviors [28]. In hierarchical networks, there are clusters of connections within clusters of connections at different scales. Network models with these properties have proved valuable in understanding the dynamical processes in those networks, which include the autonomous system level of the internet [29], networks of collaborators in open-source projects [30], and the electrical power grid [31]. What has not been previously studied is how positive or negative interactions between these hubs in scale-free networks and between the clusters in hierarchical networks influence the dynamics of those networks. In this report, we studied how positive or negative interactions between hubs and clusters influence the dynamics of those networks. Because of the known existence of such hubs and clusters in many different networks, our work here provides valuable new information on the dynamical properties that are important in the function of many different physical, biological, and social networks.

Of particular interest are studies on evolutionary games and cooperation/competition in networks, where interaction rules are compatible with different strategies, and the role of the topological properties of networks is suggested to be determinant for the evolution of systems [32,33,34,35]. Very recently, cooperation/competition dynamics have been considered in the context of multilayer networks and other configurations, including so-called higher-order interactions [10,36,37,38].

Based on the ideas developed by Deutsch [39,40], a cooperative interaction between two interacting individuals refers to the reaction with the same action by both of them (e.g., both attack each other or both support each other), while a competitive relationship refers to opposite reactions by both individuals. Subsequent mathematical studies on conflict dynamics have proposed the use of models with non-linear interactions and their generalizations to N actors arranged in a network. For example, in ref [8], the dynamic consequences of *N*-actors cooperation/competition in a small-world network were explored, starting from the generalization of the Liebovitch et al. [41] two-actor conflict model. Other reports, involving cooperation on graphs, have studied the mechanisms for the emergence of cooperating actors in scale-free networks [34], cooperation in the prisoner’s dilemma [35], and robustness of cooperation in scale-free networks [42].

Several studies focusing on the dynamics of social interactions have addressed the problem of conflict dynamics, ranging from approaches based on few-member interactions to configurations with complex topology. However, very little has been explored or theorized about the relevance of negative (or positive) interactions in scale-free networks, where hubs may have a preferential pairing tendency with other highly connected or low-degree nodes, and in configurations with a hierarchical structure. It is also important to know the role of hubs when they follow mostly cooperative or competitive behavior, and how they can become determinant for the global evolution of the system. Additionally, it has been recognized that in social interactions, the percentage of cooperators and competitors is not entirely clear as it is likely to show changes over time; however, several empirical studies have reported that the presence of cooperation is higher than that of competition [5], but there is no complete explanation for assuming a certain specific percentage. In the context of our study, it would be important to determine the dynamic consequences of changes in these percentages and their relationship to alterations in mixing patterns and network topology.

In this work, we focus on the two-actor conflict model of Liebovitch et al. [41]. In particular, networks with scale-free (SF) topologies are used to evaluate the cooperative–competitive behaviors of actors when the two-actor model is generalized to the *N*-actors model. We studied two types of networks: scale-free networks [43] with a power-law degree distribution of connections $P_{i}∼k_{i}^{-\gamma }$ and hierarchical networks [44] with a self-similar reproduction of larger patterns at smaller scales. Both of these types of networks have hubs that have a large number of connections, while the majority of nodes have a few connections, i.e., a high number of interactions are dominated by the presence of hubs. Our goal is to determine how the dynamics and the final steady-state values of the nodes of these networks depend on how these hubs are connected to each other and to the majority of the less connected nodes. For the scale-free networks, we studied the cases where there are positive correlations between the hubs, negative correlations between the hubs, and no correlations between the hubs [45,46]. We call networks with these positive correlations between hubs “assortative”, for example, social networks where celebrities are more likely to connect to other celebrities. We call networks with these negative correlations between the hubs “dis-assortative”, for example, social networks where celebrities are more likely to connect to their fans. For the hierarchical networks, we studied cases where the average clustering coefficient varies as a function of degree (number of neighbors). For these cases, the size of the rich-club clusters increases until all the hubs form a single rich-club that covers the entire network. For all of these cases, for both the scale-free and hierarchical networks, we numerically determined the dynamics and the final actor’s states as a function of the fraction of cooperative and competitive actors.

Our findings reveal that for scale-free networks, the separation between the values of the nodes is smaller when there is a balance between competitors and cooperators, while greater divergence occurs for the asymmetric cases. Where competitive interactions dominate, displays lower dispersion compared to the cases where cooperative interactions dominate. In hierarchically structured networks, our findings reveal that cooperation between the most connected actors leads to greater separation between the final states in the system, while competition between them favors a less dispersed configuration. The paper is organized as follows. In Section 2, the methods and network models are described. The results are presented in Section 3. Finally, a discussion and some concluding remarks are given in Section 4.

## 2. Methods and Models

In this paper, we use the generalization of the two-actor conflict model [41] to the *N*-actors conflict model [8], which is given by

$$\frac{dx_{i}\left(t\right)}{dt}=-m_{i}x_{i}\left(t\right)+\sum_{j=1}^{N}A_{ij}c_{ij}\tanh\left(x_{j}\left(t\right)\right),\;1\leq i\leq N,$$

where $x_{i}\left(t\right)$ represents the state of actor *i* at time *t*, *N* is the network order, $m_{i}>0$ is the inertia to neutral state 0 (conflict-free state), *A* is the network adjacency matrix, $c_{ij}$ is the strength of the feedback that actor *i* receives from actor *j*, and tanh is the hyperbolic tangent function, see [41] for the rationale for selecting $c_{ij}\tanh\left(x_{j}\left(t\right)\right)$ to depict the exerted feedback by actor $x_{j}$ on actor $x_{i}$.

The above model considers that the first term on the right-hand side of the equality completely describes the auto-dynamic dependence of an isolated actor; for this reason, we do not use self-loops (i.e., $A_{ii}=0$, 1≤i≤N), for the development of this work.

Given two interacting actors, *i*, *j* (i.e., when $A_{ij}\neq 0$), based on the ideas developed by Deutsch [39,40], we define the interaction between these two actors as (1) cooperative, if $c_{ij},c_{ji}>0$; (2) competitive, if $c_{ij},c_{ji}<0$; and (3) mixed, if $c_{ij}=-c_{ji}$. That diversity of interactions between each of the pairs of players is assigned by varying the percentage of positive entries (parameter $p^{+}$) in the network interaction matrix, *C*, with *C* satisfying (1) $C_{ij}=0$ if and only if $A_{ij}=0$; and (2) if $A_{ij}\neq 0$, then $C_{ij}=c_{ij}$. Thus, if $p^{+}=0$, it means that all interactions in the network are competitive, whereas if $p^{+}=100$, it means that all interactions in the network are cooperative. In a complementary way, we define the percentage of negative interactions as $p^{-}=1-p^{+}$.

In this work, the interactions between actors are exclusively of strong feedback, i.e., $m_{i}<\left|c_{ij}\right|$, 1≤i,j≤N, because in [41] it was seen that this type of interaction caused the actors to evolve to different states and not only to the neutral state [47]. We set $m_{i}=0.9$ and $|c_{ij}|=5$; the conditions $m_{i}$ and $c_{ij}$ being equal to constant values mean that all actors in the network experience the same inertia to state changes and the actors influence each other proportionally, respectively.

The time evolution of the states of the actors is obtained by using 4th-order Ruge–Kutta numerical integration in 1000 time steps, where the initial values are considered to be uniformly distributed within the interval (−1, 1); this implies similar amounts of positive and negative actors at the initial time, $t_{0}$.

### 2.1. Network Models and Metrics

#### 2.1.1. Scale-Free Networks with Degree Correlations

In many real systems, the description of the connectivities is given by a power-law/scale-free type distribution. A representative model for generating a scale-free network is the Barabási–Albert model, which consists in considering a set of nodes with certain links; new nodes are joined according to the preferential attachment process: nodes with higher connectivity are more likely to link new nodes [43]. The obtained network thus has power-law distribution $P∼k^{-\gamma }$ where the exponent, γ, characterizes the network. A more robust model for generating scale-free networks with a random mixture and a definite exponent is the Molloy–Reed algorithm on a set of *N* nodes [48,49,50]. Briefly, $k_{i}$ copies are generated for each node *i*, where the probability of having a degree equal to $k_{i}$ satisfies $P_{i}∼k_{i}^{-\gamma }$. These copies of the nodes are randomly linked, without repeating links and avoiding self-loops.

**Figure 1:**
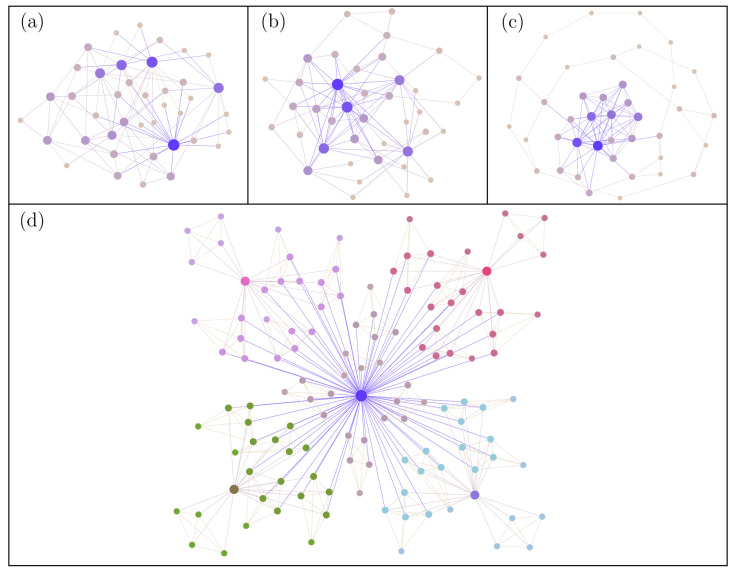
Representative cases of scale-free networks in our study. We show the cases of (**a**) dis-assortative network, (**b**) neutral network, (**c**) assortative network, and (**d**) deterministic Ravasz–Barabási network.

In some complex networks, high-degree nodes predominately connect to other high-degree nodes, such as celebrities in social media preferentially connecting to other celebrities, with these networks being described as assortative mixing. In contrast, in other networks, high-degree nodes tend to connect with low-degree nodes, such as celebrities in social media preferentially connecting only to their fans; the network is said to have a dis-assortative mixture. To capture these trends in degree–degree correlations, the parameter *r* has been proposed [45], defined as the Pearson correlation coefficient of degree between pairs of connected nodes, which can take values within the interval 1≤r≤−1. For r>0, the network shows assortative mixing patterns, while for r<0, it shows dis-assortative mixing trends. For practical purposes, the procedure introduced by Doyle et al. [51] was used to generate three different configurations: (i) assortative, (ii) dis-assortative, and (iii) neutral [22] (see Figure 1a–c). Briefly, the nodes are sorted from the highest to the lowest degree. Then, to generate case (i), we first consider the node with the highest degree and then add links to the nodes in descending order until we complete the degree of the rest of the nodes. In the opposite way, to generate case (ii), we start from the node with the highest degree and add, first, the links to the nodes of lower degree according to the ascending order. Finally, to generate the case (iii), nodes are randomly linked according to the degree of each node. In all cases considered, repetition of links and disconnectedness of the network will be avoided.

#### 2.1.2. Hierarchical Networks

To generate a network with a scale-free hierarchical structure, we use the model proposed by Ravasz and Barabási [44]. Briefly, the first step consists of generating a first cluster or complete network of five nodes; then, four replicas are created and, finally, four nodes from each cluster of replicas are connected to a node (hub) of the first cluster; this results in a network of 25 nodes, including a main node. The next step consists of replicating the first step four more times and then connecting the resulting 16 peripheral nodes to the hub node proposed in the first step; the resulting network thus consists of 125 nodes and 5 hubs. This described algorithm can be repeated, with each step increasing the number of nodes by a factor of 5. In this work, 5 similar replications were generated to obtain a hierarchically structured network with 625 nodes (see Figure 1d).

In addition, we incorporate the rich-club property, to detect the tendency that highly connected nodes tend to connect with other well-connected nodes. For this purpose, we consider a η probability that the main hubs are connected to each other in the hierarchical model described above. Thus, when η=1, all major hubs are connected, forming a rich club.

#### 2.1.3. Metrics

We list the used metrics to characterize the final configurations of the actor’s states. In all cases, *N* represents the number of actors, and brackets indicate the average of *s* independent realizations.

- The average of the absolute values of the final states is defined as
  (1) $$\bar{\left|x\right|}=\frac{1}{N}\sum_{i=1}^{i=N}\left|x_{i}\right|,$$
  where $|x_{i}|$ is the absolute value of the final state of actor *i*.
- The distance according to sign is defined as
  (2) $$L=|{\bar{x}}^{+}-{\bar{x}}^{-}|,$$
  where ${\bar{x}}^{+}$ (${\bar{x}}^{-}$) represents the average of all positive (negative) actor’s states.
- The mean global distance, *D*, is defined as
  (3) $$D={\langle \frac{2}{N(N-1)}\sum_{i=1}^{N}\sum_{j\geq i}^{N}\left|x_{i}-x_{j}\right|\rangle }_{s},$$
  where $x_{i},x_{j}$ are the final states of the actors i,j, respectively.
- The normalized range of the number of negative and positive nodes, δ, is defined as
  (4) $$\delta ={\langle \frac{|N^{+}-N^{-}|}{N}\rangle }_{s},$$
  where $N^{+}$ ($N^{-}$) is the total number of positive (negative) final states.

## 3. Results

### 3.1. Scale-Free Networks with Degree–Degree Correlations

**Figure 2:**
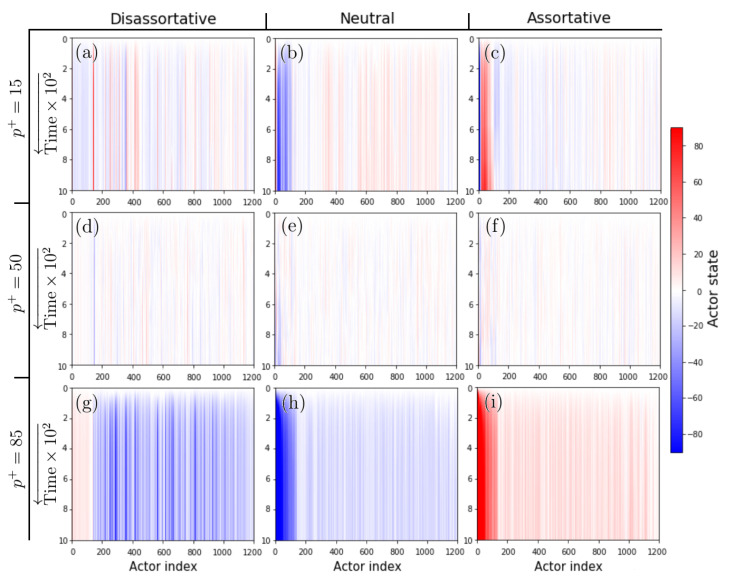
Time evolutions of the states of the actors for different levels of assortative mixing of scale-free networks. We show the cases of (left column) dis-assortative, (center) neutral, and (right column) assortative mixing. Each row represents the case of percentage of positive inputs (top row) $p^{+}=15$, (middle row) $p^{+}=50$, and (bottom row) $p^{+}=85$. We observe that for the case when most of the interactions are competitive, the system evolves towards configurations where the nodes (actors) tend to be stable but opposite states (panels (**a**–**c**)), which is mostly noticeable in the dis-assortative case (panel (**a**)). In contrast, for the case when most of the interactions are cooperative (panels (**g**–**i**)), the system evolves to reach greater amplitudes where the states of the actors tend to be markedly similar to each other, and the hubs (which correspond to small indices on the horizontal axis) are the ones that reach the greatest amplitude, except in the dis-assortative case (panel (**g**)), where the amplitude is lower. For the case with a similar presence of cooperative and competitive inputs, the system is characterized by the formation of unstable clusters for neutral configurations (panel (**e**)), while in the assortative and dis-assortative cases, these clusters are more stable (panels (**d**,**f**)). In all the simulations of this figure, we set γ=2.125.

First, we performed simulations to evaluate the changes in the time evolution for different fractions of cooperative (or competitive) entries, several values of the γ-exponent, and three configurations of degree correlations. Figure 2 shows representative time evolutions of the system for assortative, dis-assortative, and neutral configurations, and for three specific values of the percentage of positive entries (see the figure caption (Figure 2) for a detailed description of the evolutions).

**Figure 3:**
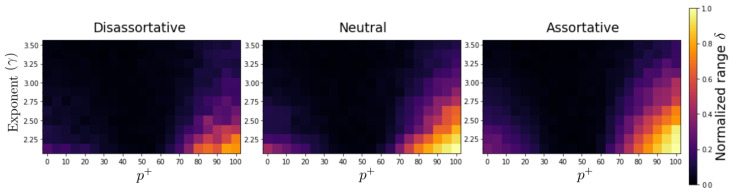
Behavior of δ in terms of $p^{+}$ and the network exponent γ. The value of δ is represented by the color bar. Each value represents the average from 20 independent realizations. In all the simulations, we considered scale-free networks with 1200 nodes.

In order to determine how the final state’s configuration is distributed, we calculated the normalized range of the number of positive and negative nodes (δ), which reflect the fact that the system can reach the cases where the states are all positive (negative) or symmetrically distributed with respect to the neutral value 0. Figure 3 presents the results of δ for three levels of assortative mixing, several values of the percentage of positive interactions ($p^{+}$), and different exponent-values γ. We observe that in the three cases (dis-assortative, neutral, and assortative), as $p^{+}$ increases, the coefficient δ decreases (reaching a minimun (around $p^{+}\approx 50$) and then increases, indicating that the state of actors achieve a symmetric distribution with respect to 0 when $p^{+}=p^{-}$ and reaches the maximum value when all inputs are cooperative ($p^{+}=100$). Moreover, as γ increases, the δ-coefficient decreases towards a more symmetric configuration (δ≈0), suggesting that the absence of large hubs favors a more balanced or symmetrical distribution of final states between positive and negative states. It is also good to note that the dis-assortative case exhibits the least imbalance, followed by the neutral case and then the assortative case. This reveals that correlations play a role in the final system configurations, with the case of positive correlations exhibiting the greatest imbalance.

**Figure 4:**
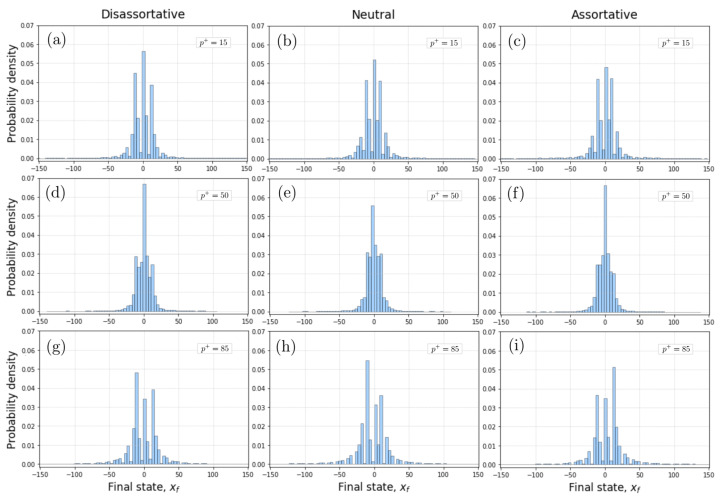
Probability density distributions (PDF) of final states ($x_{f}$) for different values of $p^{+}$ and different levels of assortative mixing of scale-free networks. (**a**–**c**) Cases of PDFs for a low value $p^{+}=15$. (**d**–**f**) Cases of PDFs for an intermediate value $p^{+}=50$. (**g**–**i**) Cases of PDFs for a high value $p^{+}=85$. Each PDF represents the results from 20 independent realizations. In all the simulations, we considered scale-free networks with 1200 nodes.

To learn how the final states are distributed, we have constructed the corresponding probability density functions, p(x), for several independent realizations shown in Figure 4. We find that p(x) changes for different mixing configurations and different values of $p^{+}$. Specifically, p(x) is considerably wider for the assortative case compared to the dis-assortative case, while in the neutral case the distribution is narrower. These results found in Figure 4, and in the context of our network model, reveal that cooperative or competitive pairwise interactions accumulate values sequentially strongly influenced by the plateaus of the hyperbolic tangent interaction function, being markedly differentiated when one type of interaction dominates, i.e., a greater amplitude for the cooperative case compared to the competitive case. It is important to note that, since Figure 4 represents the results of 20 independent realizations, some of which have more actors in positive final states and others in negative final states, the overall means of $x_{f}$ in all cases are close to zero, and the resulting p(x) display an approximate symmetry with respect to the neutral value.

**Figure 5:**
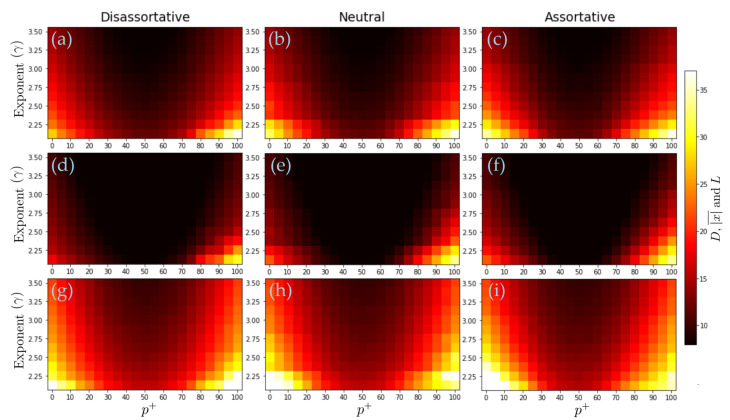
Simulation results for three distance metrics and three SF network configurations. (**a**–**c**) Behaviour of the mean global distance (*D*) for (**a**) dis-assortative, (**b**) neutral, and (**c**) assortative cases; lower values are observed in the three cases for $p^{+}\approx 50$, and these decrease even more as the exponent grows (i.e., when the hubs are reduced in size). It is noteworthy that a greater divergence is observed in the assortative case compared to the opposite case (dis-assortative). (**d**–**f**) For the average of absolute values ($\bar{\left|x\right|}$). (**g**–**i**) For distance according to sign (*L*). Each value represents the average from 20 independent realizations. In all the simulations, we considered scale-free networks with 1200 nodes.

Next, we explored the configuration of the final states of the actors in terms of the metrics described in Section 2. In particular, the final configurations are evaluated in terms of $p^{+}$, and γ for the three cases of assortative mixing. Figure 5 shows that, for a fixed value of γ, as $p^{+}$ increases, the average distance between the actors exhibits a decreasing behavior up to a minimum (around $p^{+}\approx 50$) and then increases up to a maximum, where all are cooperators. Similar behavior is observed for larger values of γ, except that the amplitudes are of lower intensity, due to the fact that hubs are becoming smaller in size. These findings indicate that the greatest divergence in the final states correspond to the cases where either all are cooperators or competitors, while the system is tighter when the population between cooperators and competitors is balanced. When the three configurations of assortative mixing are compared, we find the smallest divergence for the dis-assortative case, followed by the neutral case, while the assortative case exhibits the greatest overall mean distance (see Figure 5). Similar patterns are observed for other metrics, except for the fact that the average absolute values exhibit lower values, while the distance according to sign leads to greater values (see Figure 5).

**Figure 6:**
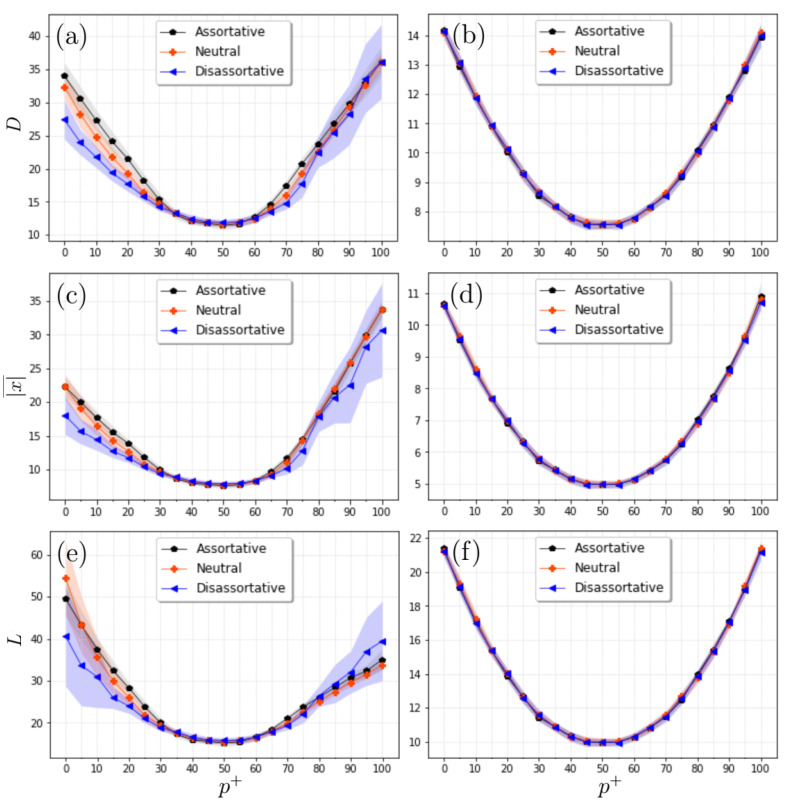
Profiles of the mean global distance (*D*), the average of absolute values ($\bar{\left|x\right|}$), and the distance according to sign (*L*) in terms of $p^{+}$ for three configurations of SF networks. We observe that, for the exponent γ=2.125 (panels (**a**,**c**,**e**)), the asymmetry of the metrics with respect to the value $p^{+}=50$ is accentuated by introducing positive correlations in the connectivities; for *D* and $\bar{\left|x\right|}$, the system is less divergent for the case where competitors dominate (panels (**a**,**c**)), and the opposite occurs for *L* (panel (**e**)). For the case when the exponent is larger (γ=3.5), the system is less sensitive to trends in the assortative pairings (panels (**b**,**d**,**f**)). Here the light-black, light-red, and light-blue regions represent the standard deviation determined from 20 independent realizations. In all the simulations, we considered scale-free networks with 1200 nodes.

For a more detailed examination of the behavior of the distance metrics as $p^{+}$ increases, the profiles of *D*, $\bar{\left|x\right|}$, and *L* are depicted in Figure 6. We show the cases of the three network configurations and two specific values of the exponent (γ=2.125 and γ=3.5). We have found that assortative mixing trends between nodes impact the distributions of the final states, with the case of positive association having the highest divergence, while less divergence is observed for the case of negative association (see Figure 6a). These results suggest that divergence in actor states can be affected by both the nature of the interaction (either cooperative or competitive) and the topology in which the actors are deployed, both of which are important for the final configurations.

Moreover, we observe that for the assortative and the neutral networks, the separation between the averages (positive and negative), measured by *L*, reflects an asymmetric behavior with respect to the $p^{+}=50$ value (Figure 6e), being larger in the case where all interactions are competitive compared to the case where all interactions are cooperative, because in the latter case, the final states tend to be either all positive or all negative, which leads to a smaller separation. The dis-assortative case tends to be more symmetric, indicating that negative degree correlations lead to a separation of mean values of the same magnitude. When the γ-exponent is larger (see Figure 6b,d,f), the separation between the curves corresponding to the different pairing trends almost disappears, most likely related to the fact that the reduction in the size of the hubs is responsible for a smaller dispersion of the actors’ states in the whole network.

### 3.2. Hierarchical SF Networks and the Rich Club

Additionally, the hierarchical structure of scale-free networks on the dynamics of the actors have been explored. We compared the final configurations coming from three cases: (i) the hierarchical network, (ii) the hierarchical network with rich club, and (iii) the random network. The rich club is built by adding links among the hubs (η=1), without any specific assignment for the sign of the entries, while the random networks are created by exchanging the links but preserving the original degree sequence. Moreover, we also consider two additional cases for the rich club: the entries of the links between hubs are all cooperative or competitive.

**Figure 7:**
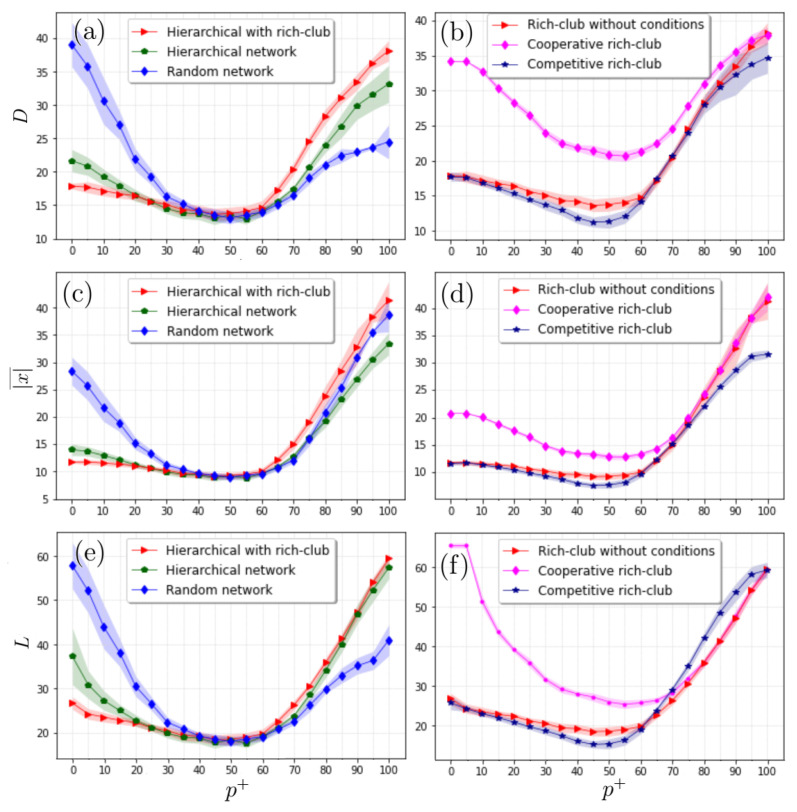
Profiles of *D*, $\bar{\left|x\right|}$, and *L* in terms of the percentage of positive entries for different configurations of hierarchical networks. We observe that the presence of a rich club alters the behaviour of the three metrics (panels (**a**,**c**,**e**)). Panels (**b**,**d**,**f**) show the behaviors of the metrics when the interactions between the hubs forming the rich club are all cooperative or competitive. We observe that cooperation between hubs significantly increases the divergence of the whole system, while competition reduces this separation between the actors. Here, the light-shaded regions surrounding each curve represent the standard deviation determined from 20 independent realizations. In all the simulations, we considered hierarchical scale-free networks with 625 nodes.

As shown in Figure 7, *D* exhibits strong dependence in terms of the percentage $p^{+}$; when most interactions are competitive, the distance is relatively low, being lowest for the hierarchical case with the rich club, followed by the purely hierarchical case, and higher values are present for the random network (Figure 7a,c,e). As $p^{+}$ increases, the three distances decay toward a minimum for intermediate values, and then the one corresponding to the rich club grows above the purely hierarchical case and the random one. This indicates that further divergence is caused by the aggregation of links when creating the rich club. Another point to note is that the hierarchical cases exhibit asymmetry with respect to the $p^{+}=50$ value that is opposite to the random case, reinforcing the fact that the hierarchical structure and links between hubs significantly impact the dynamics between actors (see Figure 7a,c,e). When comparing the cases where the rich club is entirely cooperative or competitive (see Figure 7b,d,f), the overall distances exhibit very differentiated behavior; while the cooperative case is described by relatively high distances and with symmetry with respect to the $p^{+}=50$ value, the competitive case is low and asymmetric, having similar values when $p^{+}$ is close to 100 (all cooperative).

## 4. Discussion and Conclusions

We have presented results of conflict and cooperation dynamics for a group of actors arranged in scale-free type networks, where the connectivities are dominated by a small group of hubs. Our findings reflect the importance of these link hubs, as well as the alterations when trends in degree–degree correlations are considered.

In scale-free networks, the presence of a slightly greater divergence when positive correlations are included probably reflects the fact that interactions between hubs privilege more effective feedbacks that impact hub neighborhoods. Other results reported here about scale-free networks are in agreement with previous reports about small-world networks [8], where it was found that the maximum divergence in actor values is reached when either the number of cooperative or competitive actors dominates the system. Moreover, both the results reported here about scale-free networks, and the previous results reported about small-world networks [8] found the same result, that a roughly equal mix of cooperative and competitive actors resulted in the least divergence of the values of the nodes of the network.

On the other hand, we show here that hierarchical scale-free networks have both some similarities and some differences with scale-free and small-world networks. Hierarchical scale-free networks, such as scale-free and small-world networks, show the least divergence for models where there is a roughly equal mix of cooperative and competitive actors. On the other hand, hierarchical scale-free networks, unlike scale-free and small-world networks, cause actor states to differ markedly for models dominated by cooperative actors rather than competitive actors.

Interestingly, only when the interactions in the rich-club hubs are cooperative, and not competitive, does the imbalance of either cooperative or competitive actors again increase the divergence of the values of the actors at the nodes of the networks.

Our results are relevant in the context of studies on the importance of the presence of negative or antagonistic interactions, which are considered crucial in the dynamics of social systems [5,52,53,54]. Our findings also highlight the importance of the topology where the interaction dynamics take place and highlight the fact that a balanced presence of cooperators and competitors makes the system more cohesive, compared to the case where one strategy dominates. It also raises the question of whether a similar strategy (whether cooperative or competitive) adopted by a group of connected hubs is able to guide the resulting dynamics of the whole system. This has been partially answered in our simulations, and in the context of our model, a cooperative rich-club leads to a higher divergence of the system while a competitive rich-club leads to a lower divergence. This increased system divergence, made possible through competitive interactions that increase the diversity of the system, may therefore make it more robust to external challenges.

## Data Availability

Not applicable.
